# Performance of* Clarias gariepinus* Fed Dried Brewer's Yeast* (Saccharomyces cerevisiae)* Slurry in Replacement for Soybean Meal

**DOI:** 10.1155/2017/8936060

**Published:** 2017-01-23

**Authors:** Shola Gabriel Solomon, Gabriel Arome Ataguba, Gabriel Enemona Itodo

**Affiliations:** Department of Fisheries and Aquaculture, University of Agriculture, PMB 2373, Makurdi, Benue State 970001, Nigeria

## Abstract

Following disparity of earlier results, this study tested the performance of African catfish* Clarias gariepinus* fed dried brewer's yeast slurry meal (DBYM) based diets. Fingerlings of* C. gariepinus* with pooled mean initial weight of 1.58 ± 0.01 g were stocked in hapas (1 m × 1 m × 1 m) immersed in an earthen pond at a density of 15 fish per cage. Five diets with increasing substitution of soybean meal with 25%, 50%, 75%, and 100% of dried brewer's yeast and a control without dried brewer's yeast (0% substitution) were evaluated for 8 weeks. Palatability of diets reduced with increasing levels of DBYM. Growth and utilization parameters such as weight gain, feed conversion ratio, protein efficiency ratio, and specific growth rate differed significantly (*p* < 0.05) among treated groups. Specific growth rate decreased with increasing substitution while the best feed conversion ratio was obtained in the diet devoid of DBYM. Protein efficiency and utilization decreased with increasing levels of DBYM. Body composition was also affected by inclusion of DBYM with significant differences (*p* < 0.05) being observed across the diets. The trend in body composition follows the utilization of the diets. We conclude that the optimal range of inclusion and substitution of soybean meal with DBYM in* C. gariepinus* feed is between 1% and 14% of dry matter.

## 1. Introduction

Fish farming relies on feeds produced using ingredients such as soybean, fishmeal, corn meal, rice bran, fish oil, and other vegetable oils that are also highly demanded by terrestrial animal agriculture as well as human nutrition [1]. Feed quantity is a significant variable affecting fish farming [2] because feed accounts for over 50% of production costs [1, 3]. Hence feeds which can offer low FCR matched with species that can deliver low FCR will be beneficial. However, fish feed development in Sub-Saharan Africa has not made a significant progress as expected [4]. Fishmeal still remains the ingredient of choice in fish feeds because of its amino acid profile and acceptability by fish. According to [5], the nutritional value of a feed is determined by its digestibility and ease of assimilation while its quality depends on the growth of the animal as a result of consumption of the feed.

The aquaculture of* Clarias gariepinus* is growing steadily but farmers need to cut costs incurred on feed; hence the availability of cheap feed that meets the requirement of* C. gariepinus* would go a long way to increasing profitability. According to [6, 7], the development and expansion of aquaculture depend on availability of good quality and relatively inexpensive feed ingredients for the formulation of compounded feed.

Yeast slurry is usually recovered in the process of brewing and used for repitching but some slurry that is deemed unsuitable for repitching is moved into waste storage [8]. Brewer's yeast* (Saccharomyces cerevisiae)* has been identified as an ingredient with several positive factors [9]. Brewer's yeast slurry* (S. cerevisiae)* contains about 45% protein, 1% lipid, and 2.7% of crude fibre [10]. It has an excellent amino acid profile but its shortcoming lies in the deficiency of sulphur containing amino acids such as methionine and cystine with a high content of lysine [11]. Temperature plays an important role in the quality of the slurry. High temperatures tend to damage the viability of yeast for the beer industry [12] but this is not of significance in the animal feed industry as autolytic breakdown of yeast which occurs at 50°C can be optimized for extraction of protein and nitrogen contents [13].

Yeast has been used extensively in poultry and other animals as a growth promoter and also additive to enhance fibre utilization. The fermentation of feed dough by yeast has been utilized alongside local binders to produce local feed having greater water stability and floatation and it was discovered that cassava and corn flour produced pellets with greater water stability [14]. Yeast addition in broiler chicken diets has been reported to lower FCR [15] while [16] reported that yeast supplementation is ideal at the starter phase compared to the finisher phase of broiler production. According to [17], an inclusion level of 200 mg yeast per kg of diet for broilers resulted in better feed efficiency. Furthermore, [18] reported that rabbits fed a supplemental diet of cultured* S. cerevisiae* at 1.5 g/kg diet efficiently converted feed compared to rabbits fed unsupplemented feed and at a supplementation rate of 3.0 g/kg of diet, haematological parameters were greatly improved. Earlier problems of foaming in brewer's yeast used for animal feed were overcome through a suggestion that fermentation in the beer industry is prolonged before cooling while rinsing with water was considered impracticable [19]. However, yeast washing is currently being used as a means of removing bacterial contamination from yeast [20].

Supplementation of fish feed with yeast has yielded mixed results with lower inclusion levels being favoured for most fish. A substitution level of 30% yeast has been advocated for Koi Carp [21] while 25% seems ideal for rainbow trout* (Oncorhynchus mykiss)* [22], cobia* (Rachycentron canadum)* [23], and Gilthead Sea Bream* (Sparus aurata)* [24]. Tilapia* (Oreochromis niloticus)* has been reported to utilize diets with 20% fishmeal substituted with brewer's yeast effectively with higher percentages eliciting deleterious effects on growth [25]. The digestibility of spent yeast by catfish has been reported as 35% [26]. The African catfish has been reported to produce an FCR of 0.56 with an inclusion of 4% of graded baker's yeast in the diet [27]. Furthermore, hybrid* Clarias gariepinus* have been reported to utilize diets with 2% level of dried brewer's yeast effectively with the determination of optimal levels beyond 2% inclusion being open for further investigation [28]. However, substitution of soybean meal with bioactive yeast in the diet of the African catfish at 50% level has been reported without adverse effects [29]. The current level of inclusion of dried brewer's yeast at 2% irrespective of soybean meal in the diet needs to be improved upon as suggested [28]. Previous reports on use of dried brewer's yeast did not actually include the method of drying the slurry. This study therefore aims to show a drying method and determine the boundaries for the safe inclusion of dried brewer's yeast while replacing soybean meal in the diet of the African catfish* (C. gariepinus)*.

## 2. Materials and Methods

*Clarias gariepinus* fingerlings of average weight 1.58 ± 0.005 g were obtained from a fish farm at Makurdi Nigeria and transported to the University of Agriculture, Makurdi. The fish were acclimatized for two weeks and fed with a commercial diet. Feeding with this diet was stopped one day prior to the commencement of feeding using the yeast slurry based diets. Groups of 15 fingerlings of* C. gariepinus* were randomly weighed and stocked into fifteen different hapas (1 m × 1 m × 1 m) immersed in earthen ponds.

Brewer's yeast slurry was obtained from Benue Breweries Makurdi and dried to a constant weight at 50°C in an oven. The yeast species is the top-fermenting type* (Saccharomyces cerevisiae)* with fermentation occurring at temperatures between 20 and 25°C. Fresh yeast slurry was obtained in batches from the brewery and transported in a plastic container of 10 litres capacity with ice packs surrounding it. Considering the flocculation ability of the yeast strain as seen from settling at the bottom of the transporting vessel, upon arrival at the laboratory, the liquid slurry was shaken gently by upside-down container flips to achieve homogeneity, centrifuged at 5000 rpm using a Centrikon T-324 centrifuge for 5 minutes; then the supernatant was discarded. The resulting solid residue was washed using cool (4°C) sterilized distilled water and quickly filtered using a suction filter with No. 1 filter paper. Solid residues were spread on sterilized, dried (105°C for 3 hours) aluminium trays and quickly transferred to the oven which was already preheated to 50°C. Drying was achieved in 16 hours following an earlier trial with triplicate samples to determine time of drying to constant weight. Heat treatment of yeast at 50°C promotes autolysis of the yeast cells [34]. This is the optimized temperature according to [13] for the release of *α*-amino nitrogen in the process of yeast autolysis. The choice of 50°C therefore was based on the aforementioned research. Soybean was toasted before milling to eliminate or reduce the effects of antinutritional factors and improve digestibility. Other ingredients were prepared according to laid down procedures [25]. The proximate composition of oven dried yeast slurry (Table 1) was determined using standard methods [35] and compared with values in literature.

Five diets with a target crude protein level of 35% were formulated for the fingerlings using programming facility of Microsoft Excel® with details of proximate, amino acid, and mineral compositions of the ingredients taken into consideration. Inclusion levels were determined appropriately while substitution levels for brewer's yeast slurry meal for soybean meal were varied from 0% to 100% hence: diet 1 [0%], diet 2 [25%], diet 3 [50%], diet 4 [75%], and diet 5 [100%]. Ingredient inclusion was determined on a dry matter basis and converted to weights as fed (Table 2). Amino acid requirements as obtained from literature were used to determine the chemical score for each amino acid in all five diets during formulation of the diets (Table 3). The diets were pelleted after weighing and mixing of the ingredients and dried to constant weight in an oven at 50°C for effective preservation.

Prior to the commencement of feeding, the proximate composition of fish carcass was determined using standard methods [35]. After feeding trials were stopped, proximate composition of test fish in all diets was also determined to understand the effect of feeding brewer's yeast based diets on the body composition of the African catfish.

Water quality in each hapa was monitored weekly. Dissolved oxygen was checked using standard DO meter (Hana Instruments), pH was determined with a handheld pH meter, and temperature was taken with a mercury-in-glass thermometer. Secchi disc visibility was taken every week and used as an index of water turbidity. Mean water temperature was 27.6°C, mean dissolved oxygen was 5.32 mg·L^−1^, pH averaged 7.9, and secchi disc visibility was 34.6 cm.

Gross energy values as well as the protein to energy ratio of each diet were determined after proximate composition of the diets was determined. Determination was done on the dry matter basis.

Each diet was fed to the catfish in triplicate hapas twice daily (09:00 hr, 16:00 hr) at 5% body weight for 56 days. Total fish weight in each hapa was determined every week and the amount of diet was adjusted accordingly. Growth response and feed utilization indices were estimated using various growth indices including weight gain, feed conversion ratio (FCR), specific growth rate (SGR), protein efficiency ratio (PER), and apparent net protein utilization (ANPU).

Data obtained was subjected to statistical analysis using Analysis of Variance (ANOVA) to determine if there were significant differences among the means and where differences occurred, means were separated using Fisher's Least Square Differences (LSD). The nature of data for the final weight necessitated the use of Analysis of Covariance (ANCOVA) to determine if differences exist in the weight gain using final weight as covariate. Survival rates were arcsine transformed before analysis and retransformed to percentages after analysis.

## 3. Results

The proximate composition of the diets as determined is presented in Table 4. The moisture contents were below 12%.

**Figure 1:**
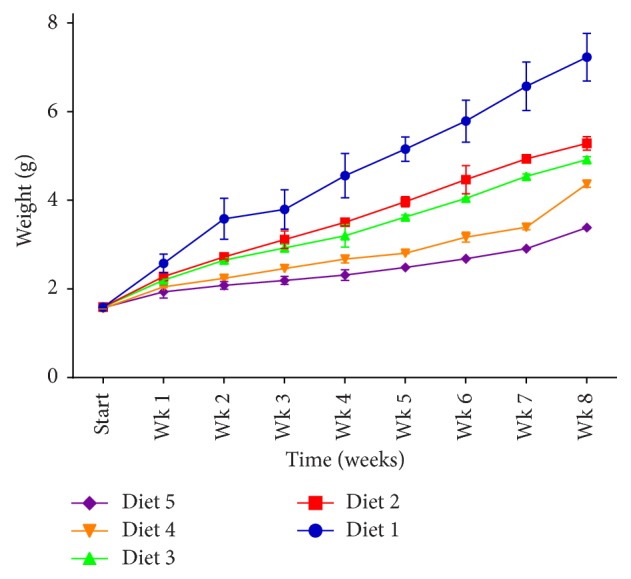
Weekly growth of* Clarias gariepinus* fed dried brewer's yeast meal substituted for soybean meal.

Weekly weight increased for the experimental fish fed diets with varying inclusion levels of dried brewer's yeast slurry meal (Figure 1). There was a uniform pattern of growth for all diets for the first week. Fish fed diet 1 increased in weight above the other treatments after week one and continued this trend until week eight. This was followed by fish fed diets 2 and 3. Fish fed diets 4 and 5 lagged behind those of diets 2 and 3 after the first week.

The growth performance and utilization of dried brewer's yeast slurry by* Clarias gariepinus* are presented in Table 5. The mean weight gain was found to vary from 1.81 g (diet 5) to 5.64 g (diet 1) with fish fed diet 1 having the highest weight gain which differed significantly (*p* = 0.001) from all other diets. Similarly, the specific growth rate varied from 01.37% per day (diet 5) to 2.70% per day (diet 1). The Feed conversion ratio differed significantly (*p* = 0.000) among the treated fish. The feed conversion ratio increased with increasing levels of dried brewer's yeast in the diets. Diet 1 produced the best FCR and diet 5 the worst. Protein use was more efficient from diet 1 and least efficient from diet 5. More protein from the feed was retained in the body by fish fed diet 2 while fish fed diet 5 retained the least protein from the feed. Feed intake by fish differed significantly (*p* = 0.003) with fish fed 100% soybean inclusion diet (diet 1) having the highest feed intake and fish fed the 100% inclusion of DBYM (diet 5) having the least feed intake and this also translates into the same pattern for protein intake which ranged from 2.55 g (diet 5) to 4.37 g (diet 1). Values for protein intake also differed significantly (*p* = 0.002).

The carcass composition of the experimental fish as presented in Table 6 shows that initial moisture level was similar across the diets but this varied significantly (*p* = 0.007) from the initial value in the fish prior to feeding with dried brewer's yeast.

Crude protein content of fish flesh decreased with increasing substitution of dried brewer's yeast with significant differences (*p* = 0.000) observed among treatments and in comparison to the initial value. Lipid content of fish flesh differed significantly among treatments and initial values. Ash content ranged from 3.09% (diet 2) to 3.32% (diet 5).

## 4. Discussion

Increasing levels of dried brewer's yeast slurry meal in the diet of* C. gariepinus* do not seem to support growth. Diet 1 with 0% DBYM in substitution for soybean meal produced the best specific growth rate as well as feed conversion efficiency. Reference [25] reported that the daily growth coefficient of tilapia reduced with increasing levels of yeast in substitution for fishmeal. Furthermore, [23] reported reduction of weight gain by cobia with increasing levels of yeast based protein in the diet. The rainbow trout* (Oncorhynchus mykiss)* does not also tolerate high levels of DBYM in its diet [22]. The inclusion of DBYM in the diet of* C. gariepinus* negatively affected SGR, FCR, and PER with a negative trend being observed beginning at 25% replacement of soybean with DBYM. According to [21], replacement of fishmeal at 40% level and above by DBYM in the diet of Koi Carp* (Cyprinus carpio)* led to reduced weight gain, SGR, PER, apparent digestibility, and increased FCR. At inclusion levels ranging from 0% to 2%,* C. gariepinus* has been reported to effectively utilize diets with FCR reducing as inclusion rate increased [28]. This therefore suggests that the substitution/inclusion rate range as used in the present study is wide and with the results, the range is narrowed to between 0 and 25% substitution for soybean meal.

Reduced palatability of the feed with increase in inclusion of DBYM was reflected in the growth response of* C. gariepinus* (Figure 1). Survival (Table 5) was unaffected by consumption of the diet. This is however at variance with reports by [23, 29]. Furthermore, [29] reported that bioactive yeast can replace soybean in the diet of* C. gariepinus* up to 50% level with acceptability increasing to this point and total mortality occurring when soybean is replaced totally by yeast. On the other hand, [36] reported that survival of juvenile beluga* (Huso huso)* was not affected by dietary inclusion of brewer's yeast at 2% level.* Clarias gariepinus* as observed did not utilize DBYM well at 25% substitution for soybean meal when compared with 100% soybean meal inclusion. Although live yeast has been reported to be effective in boosting immune response of fish, there was no report on difference in feed intake between live and inactive yeast [37–39]. The present results show that feed intakes reduce significantly with DBYM at high inclusion levels but drying of yeast has been proven to also enhance immunoactivity through increased *β*-D-glucan levels [40]. Diets of* C. gariepinus* that are prepared with soybean meal being substituted with DBYM will therefore be ingested more at low levels of substitution that lie between 0 and 25% (i.e., 0 to 14.5% dry matter inclusion of DBYM).

Feed intake reduced as inclusion of DBYM increased. Reference [22] had reported feed expulsion from the mouth upon ingestion by rainbow trout fed 50% and 75% brewer's dry yeast diets. The PER of fish fed DBYM reduced with increasing levels of DBYM except for 75% level of substitution which increased with a decrease at 100% DBYM substitution. This undulating trend suggests that proteins from DBYM may contain or lack certain factors that lead to diet rejection which according to [22] is due to the presence or absence of certain dietary factors in yeast meal. The biochemical composition of yeast suggests it has 10 to 15% nucleic acids in the protein [37]. Hence, intake of yeast increases concentration of nucleic acid particularly purine nucleotide in the gut with attendant uric acid accumulation induced by production of lactate [38] which negatively affects protein metabolism [37]. Intake of nucleotides by Atlantic salmon has been found to impact the gastrointestinal system by increasing gut surface areas well as fold height of the intestine [39]. However, the operation of nuclease, an enzyme responsible for the digestion of nucleotides, is poorly understood in fish [40].

Protein conversion was better for fish fed higher substitutions of DBYM for soybean although fish fed these diets did not take as much feed as the other treatments. The intake of feed inevitably affected the intake of protein and hence efficiency of protein although conversion was high. In addition, the fact that yeast is deficient in the sulphur containing amino acids methionine, lysine, and cystine also contributes to poor protein efficiency. The chemical score for cystine in the diets reduced with increasing levels of DBYM inclusion; hence poor performance of diets occurred with higher inclusion levels (>25%) of DBYM. Substitution of soybean meal at 25% with DBYM still produced a chemical score of 100 for cystine; hence the performance of this inclusion is superior compared to the rest. Methionine is converted to cystine if there is a short supply of cystine in the diet [41]; hence the 40–60% methionine sparring ability of cystine [42, 43] is reversed and methionine deficiency occurs. Cysteine can provide about 50% of the needs of total sulphur amino acids in fish [44]. This can explain why there is poor protein efficiency but high protein conversion. Low level inclusion of yeast in the diet of* C. gariepinus* does not seem to affect protein metabolism as productive protein value or ANPU and PER were reported to increase with increasing levels (0%, 1%, 1.5%, and 2%) of yeast of the diet [28].

Whole body composition of* C. gariepinus* was affected by substituting yeast for soybean meal. However, a report by [45] shows that supplementing diets of hybrid striped bass have no significant effect on body composition. Similarly, [46] reported that the crude protein of rainbow trout flesh did not differ with increasing supplementation of yeast RNA extract. Reference [25] reported that the body composition of tilapia was affected by supplementing the diet with yeast; hence there was a reduction in body protein that was attributed to poor amino acid profile of the diets. Reference [23] presented a similar scenario to the one obtained for* C. gariepinus* here with differences being observed in muscle protein as substitution of fishmeal with yeast increased but substitution at 25% and total inclusion of fishmeal at 100% did not produce any significant effects on muscle protein. Similarly, [47] reported significant differences in the crude protein composition of* M. rosenbergii* postlarvae fed yeast supplemented diets. This similarity notwithstanding, the authors reported increasing crude protein levels in the flesh of the prawn with increasing yeast supplementation as against the declining trend observed for* C. gariepinus*. This clearly shows that prawns can tolerate yeast as a dietary ingredient.

On the whole, substitution of soybean meal with dried brewer's yeast meal in the diet of* C. gariepinus* between ~14.5 and 58% dry matter did not produce any significant advantages over the use of soybean at full inclusion. It is clear that substitution and inclusion levels for optimal performance are between 1% and 14% of dry matter.

## Figures and Tables

**Table 1:** The proximate composition of brewer's yeast slurry *(Saccharomyces cerevisiae)*.

Parameter	% composition		
	Brewer's yeast meal [30]	Liquid brewer's yeast [31]	Present analysis^*∗*^
Moisture	7.6	85.6	7.6 ± 0.15
Protein	46.1	7.6	45.9 ± 0.25
Lipid	1.3	0.1	1.7 ± 0.19
Ash	8.1	1.4	8.3 ± 0.12
Fibre	2.9	0.7	2.7 ± 0.06
NFE	34	4.6	33.8 ± 0.17

^*∗*^ *n* = 3.

**Table 2:** Inclusion level of ingredients in prepared diets.

Ingredient	Diet 1		Diet 2		Diet 3		Diet 4		Diet 5	
	% dry matter	g/kg feed mix	% dry matter	g/kg feed mix	% dry matter	g/kg feed mix	% dry matter	g/kg feed mix	% dry matter	g/kg feed mix
Fish meal	11	112.24	9.50	97.21	8.00	81.73	6.40	65.01	4.80	48.29
SBM	57.8	562.99	43.35	417.11	28.90	273.55	14.45	134.02	0	0.00
BYS	0	0	14.45	144.02	28.90	273.55	43.35	428.92	57.8	566.43
Corn meal	26.20	278.81	27.70	295.60	29.20	311.11	30.80	326.29	32.4	339.96
Vit. premix	2.00	18.37	2.00	18.42	2.00	18.39	2.00	18.28	2	18.11
Min. premix	2.00	18.41	2.00	18.44	2.00	18.43	2.00	18.33	2	18.15
Salt	0.50	4.59	0.50	4.61	0.50	4.60	0.50	4.57	0.5	4.53
Oil	0.50	4.59	0.50	4.61	0.50	4.60	0.50	4.57	0.5	4.53

**Table 3:** Amino acid requirements and chemical score of prepared diets.

Amino acid	Requirements (% protein) [32, 33]	Chemical score				
		Diet 1	Diet 2	Diet 3	Diet 4	Diet 5
Arginine	4.3	158.97	154.17	148.41	142.66	136.90
Histidine	1.5	147.67	144.79	140.87	137.10	133.32
Isoleucine	1.56	301.68	295.15	286.01	276.88	267.74
Leucine	4.87	161.05	162.17	162.11	162.08	162.05
Lysine	4.49	142.34	154.34	165.33	176.31	187.31
Methionine	1.15	154.98	149.50	142.19	134.67	127.14
Cystine	1.15	121.81	101.34	79.53	57.77	35.97
Phenylalanine	2.5	187.88	184.71	180.06	175.45	170.83
Tyrosine	2.5	137.36	140.11	141.76	143.43	145.10
Threonine	2.04	200.08	203.32	204.98	206.58	208.17
Tryptophan	0.5	249.51	245.59	239.60	233.70	227.79
Valine	2.08	241.52	251.17	258.55	265.98	273.41

**Table 4:** Proximate composition of diets with dried brewer's yeast meal (DBYM) in replacement of soybean meal (dry matter basis).

Parameters	Diet 1	Diet 2	Diet 3	Diet 4	Diet 5	*p* value
Moisture	11.38 ± 0.04^a^	11.02 ± 0.05^b^	10.90 ± 0.03^b^	10.66 ± 0.12^c^	10.42 ± 0.01^d^	0.000
Protein (%)	34.50 ± 0.06^e^	35.30 ± 0.34^d^	36.23 ± 0.10^c^	37.07 ± 0.06^b^	37.83 ± 0.14^a^	0.000
Lipid (%)	7.16 ± 0.01^a^	6.20 ± 0.10^b^	5.29 ± 0.03^c^	4.40 ± 0.06^d^	3.48 ± 0.03^e^	0.000
Ash (%)	9.18 ± 0.01^c^	9.36 ± 0.07^b^	9.51 ± 0.05^b^	9.74 ± 0.07^a^	9.89 ± 0.03^a^	0.000
Fibre (%)	5.59 ± 0.04^a^	4.89 ± 0.03^b^	4.17 ± 0.11^c^	3.44 ± 0.01^d^	2.75 ± 0.11^e^	0.000
NFE (%)	43.58 ± 0.09^d^	44.26 ± 0.32^c^	44.80 ± 0.25^bc^	45.36 ± 0.09^b^	46.05 ± 0.09^a^	0.000
Gross energy (KJ)	15.77	15.59	15.56	15.46	15.35	—
P : E ratio (mg/KJ)	21.88	22.65	23.29	23.98	24.64	—

Means (*n* = 3) in the same row with different superscripts differ significantly (*p* < 0.05).

**Table 5:** Nutrient utilization of *Clarias gariepinus* fed dried brewer's yeast meal substituted for soybean meal for 8 weeks.

Parameters	Diet 1	Diet 2	Diet 3	Diet 4	Diet 5
Initial Wt	1.59 ± 0.01	1.59 ± 0.01	1.59 ± 0.01	1.56 ± 0.01	1.57 ± 0.01
Final Wt	7.23 ± 0.54^a^	5.28 ± 0.15^b^	4.92 ± 0.07^b^	4.37 ± 0.07^b^	3.38 ± 0.07^c^
Wt gain	5.64 ± 0.52^a^	3.69 ± 0.14^b^	3.33 ± 0.06^b^	2.80 ± 0.08^b^	1.81 ± 0.03^c^
SGR	2.70 ± 0.12^a^	2.14 ± 0.04^b^	2.02 ± 0.01^bc^	1.84 ± 0.04^c^	1.37 ± 0.02^d^
FCR	2.24 ± 0.02^c^	2.69 ± 0.02^b^	2.78 ± 0.01^b^	2.76 ± 0.06^b^	3.74 ± 0.13^a^
PER	1.29 ± 0.01^a^	1.05 ± 0.01^c^	0.99 ± 0.00^c^	1.21 ± 0.03^b^	0.71 ± 0.02^d^
ANPU	3.95 ± 0.34^d^	4.89 ± 0.16^c^	4.78 ± 0.10^c^	6.83 ± 0.03^a^	6.11 ± 0.12^b^
IFI (g)	12.66 ± 1.07^a^	9.92 ± 0.32^b^	9.26 ± 0.19^bc^	7.72 ± 0.04^cd^	6.75 ± 0.14^d^
IPI (g)	4.37 ± 0.37^a^	3.50 ± 0.11^b^	3.35 ± 0.07^b^	2.32 ± 0.01^c^	2.55 ± 0.05^c^
Survival^*∗*^	80.43 ± 0.71	90.25 ± 0.32	90.25 ± 0.32	98.30 ± 1.71	98.30 ± 1.71

Means (±SEM, *n* = 3) in the same row with different superscripts differ significantly (*p* < 0.05).

^*∗*^Percentage survival data were arcsine-square root transformed, analysed, and back transformed as percentages.

FI = feed intake (dry matter) and PI = protein intake (dry matter).

**Table 6:** Body composition of *C. gariepinus* fed diets containing various amounts of dried brewer's yeast substituted for soybean meal for 8 weeks.

Parameters	Moisture	Protein	Lipid	Ash
Initial	75.60 ± 0.03^a^	15.03 ± 0.00^e^	6.21 ± 0.02^ab^	3.17 ± 0.02^bc^
Diet 1	74.45 ± 0.15^b^	17.13 ± 0.02^a^	5.31 ± 0.13^c^	3.11 ± 0.04^c^
Diet 2	74.80 ± 0.10^b^	17.11 ± 0.02^a^	5.01 ± 0.09^c^	3.09 ± 0.03^c^
Diet 3	74.75 ± 0.15^b^	16.01 ± 0.03^b^	6.02 ± 0.14^b^	3.23 ± 0.02^b^
Diet 4	74.50 ± 0.20^b^	15.86 ± 0.03^c^	6.52 ± 0.25^a^	3.12 ± 0.02^c^
Diet 5	74.73 ± 0.09^b^	15.60 ± 0.01^d^	6.36 ± 0.06^ab^	3.32 ± 0.02^a^

Means (±SEM, *n* = 3) in the same column with different superscripts differ significantly (*p* < 0.05).
